# Density and Diversity of Microbial Symbionts under Organic and Conventional Agricultural Management

**DOI:** 10.1264/jsme2.ME18138

**Published:** 2019-06-13

**Authors:** Orsolya Gazdag, Ramóna Kovács, István Parádi, Anna Füzy, László Ködöböcz, Márton Mucsi, Tibor Szili-Kovács, Kazuyuki Inubushi, Tünde Takács

**Affiliations:** 1 Institute for Soil Sciences and Agricultural Chemistry, Centre for Agricultural Research, Hungarian Academy of Sciences Herman Ottó u. 15, Budapest Hungary; 2 Department of Plant Physiology and Molecular Plant Biology, Eötvös Loránd University H 1117, Pázmány Péter sétány 1/C., Budapest Hungary; 3 Graduate School of Horticulture, Chiba University Matsudo, Chiba Japan

**Keywords:** agroecology, arbuscular mycorrhizal fungi, genetic diversity, organic farming, *Rhizobium*

## Abstract

The influence of organic and conventional farming and agroecology on the diversity and functioning of indigenous soil microbial communities was examined using a multifactorial analysis based on an extended minimum data set of classical status and functional tests. Main soil physicochemical properties and selected microbiological indicators, the quantity of heterotrophic or aerobic spore-forming bacteria, basal and substrate-induced respiration, catabolic activity with MicroResp^™^, and fluorescein diacetate enzyme activity were characterized. A pot experiment applying the most probable number method was designed with soil dilution series using *Pisum sativum* L. and *Triticum spelta* L. to assess the symbiotic infectivity and genetic diversity of key indicator groups of the plant microbiome, *e.g*. nitrogen-fixing bacteria (rhizobia) and arbuscular mycorrhizal fungi. Soil pH, humus content, CFU, enzyme activity, and soil respiration were significantly higher in organic soils. The activity of soil microorganisms was mainly related to clay, humus, calcium, and magnesium parameters. A redundancy analysis test of catabolic activities showed that samples were grouped according to different substrate utilization patterns and land uses were also clearly separated from each other. Farming practice influenced the abundance and diversity of microbial populations. Dark septate endophytic fungi were only found in conventional soils. In addition to confirming soil health improvements by organic management, our results highlight the importance of a complex evaluation including both classical status and functional parameters of soil microbiota, which may more reliably indicate a shift in the quality status of soils.

Historically, soil quality or its synonym, soil health had been defined as the capacity of soil to support agricultural productivity and fertility in agroecology (86). However, healthy soils are essential for all terrestrial–including agricultural ecosystems to be resistant, resilient, and to have functionally redundant responses to different disturbances. Therefore, soil quality was redefined as the functional capacity of soils to establish optimal conditions for living organisms and human purposes (21, 42), becoming also increasingly relevant as soil ecosystem services (28). The most critical point of this approach is the identification of relevant indicators for soil health and sustainability. In agriculture, sustainability is frequently associated with minimal soil chemical inputs and effective nutrient recycling, for example, nutrient supply and decom-position (70), amongst which soil microbiological properties, such as diversity, functionality, resilience, and tolerance, play an important role (4, 28, 38).

Recent studies have focused on elucidating the dynamic relationship between agricultural practices and soil biological processes in order to support functional biological and ecological cycles and develop sustainable farming systems. Depending on its intensity, soil management may either increase or suppress the diversity and activity of soil microorganisms. Organic farming systems depend predominantly on the soil nutrient resources provided by the soil microbiota as opposed to the high-input management of conventional agricultural systems (9, 71). Therefore, research on microbial indicators needs to cover (I) the relationship between genetic and functional biodiversity, (II) the potential parameters for monitoring activities that help to predict the responses of different ecosystems to global changes, and (III) the indicators that may be used to establish models and optimal sampling strategies (55). The following groups of biological indicators have been proposed for use in soil quality assessments: I, activity rates and functional diversity, II, biomass, and III, community structure (55, 67, 73). Technical (infrastructural) and methodological developments provide the opportunity to examine soil physical, chemical, and biological characteristics, which need to be selected wisely to create a minimum data set (MDS), a well-known approach to characterize soils and evaluate soil quality (8, 71).

Symbionts, such as arbuscular mycorrhizal fungi (AMF), bacteria, including rhizobia, and other plant growth-promoting species are key indicators due to their elemental influence on the functioning of agricultural ecosystems. The nitrogen (N) input of soils is based on symbiotic biological N_2_ fixation (BNF), which may also be the main source in organic farming (77). Although research on the plant microbiome is increasing due to novel molecular and analytical methods (85), limited information is currently available on the long-term changes in natural bacterial populations in relation to host plant diversity and the abundance of rhizobia (28, 73).

The role of AMF in plant vitality and fitness has been well documented (75). AMF principally promote mineral nutrient acquisition (31, 76) and water uptake (10) by host plants from soil; however, several other beneficial effects of AMF have been described (59). Although increased phosphate uptake appears to be the most relevant advantage of AMF on plants (76), the improved uptake of other nutrients, such as N, zinc (Zn), copper (Cu), and potassium (K), may also be involved in direct mycorrhizal effects (31). Plant growth-promoting symbiotic rhizobacteria (PGPR) are more abundant in the mycorrhizosphere than in soil without an AMF hyphal network (13). Although AMF are not host-specific, some preference is expected (14). In the case of low input, sustainable agricultural management, indigenous AMF, and PGPR are very beneficial for plant health and production; hence, the stability of an organic farming system may be predicted by its diversity, infectivity, and functionality (33).

Dark septate endophytes (DSE) represent a polyphyletic, ecologically, and physiologically variable root-colonizing group of fungi that are abundant in several habitats worldwide (2, 40, 41, 54). DSE are characterized by the presence of darkly melanized, intra- and intercellular septated hyphae, or the microsclerotia of ascomycetous fungi in roots (44, 68). DSE have weak host or habitat specificity, and the root samples of numerous plant species collected from arctic to tropical ecosystems are frequently colonized by them (68). Their function remains ambiguous (39, 44).

Our aim was to perform a multifactorial analysis of the effects of agricultural practices on the diversity and function of indigenous microbial communities. Numerous chemical and physical soil properties, microbiological indicators, and their correlations were investigated to evaluate the quality of differently managed soils. Microbial abundance was investigated in the organic farming (OF) and conventional farming (CF) systems of a long-term field experiment in calcic chernozem soil (Martonvásár, Hungary). Their functional diversity was characterized by the catabolic activity patterns of indigenous soil microbes using the MicroResp^™^ method.

Community level physiological profiles (CLPP) are most commonly analyzed by the Biolog^®^ (29) and MicroResp^™^ methods (17). The Biolog^®^ method is a rapid analysis (36, 63), although only a fraction of the microbiome is analyzed due to soil extraction before the reaction. Furthermore, selective carbon sources may change bacterial community structures during the incubation period. The total potential and *in situ* activity of soil microbes are measured by the Biolog^®^ and MicroResp^™^ methods, respectively, based on their different incubation times (minimum 24 h for Biolog^®^, 5–6 h for MicroResp^™^). Due to its high sensitivity, MicroResp^™^ is a widespread method in the field of biomonitoring (18, 20, 53, 78), which may discriminate between the soil microbial communities that the Biolog^®^ system is not appropriate for (56).

In a pot experiment implementing the most probable number (MPN) test, the microbiota of a tripartite (host plant-bacteria-AM fungi) symbiotic system was isolated from both farming systems. The infectivity and molecular diversity of nitrogen-fixing rhizobia and AM fungi were investigated in pea (*Pisum sativum* L.) and dinkel spelt wheat (*Triticum spelta* L.) roots.

## Materials and Methods

### Characterization of experimental fields

Soil samples were collected in May 2012 from the organic and conventional plots of a 15-year-old field experiment in the Centre for Agricultural Research, Martonvásár (Hungary) with a calcic chernozem soil (23, 24) (Table S A1), with the following GPS coordinates: 47° 18′ 38″ N, 18° 46′ 45″ E. The upper 20 cm of soil was sampled from 12 different points along the diagonal of a 0.5-ha field (*n*=12 for both OF and CF). Homogenized samples were air-dried, ground, and sieved through a 2-mm mesh for physical and chemical analyses. A total of 0.5 kg of soil samples from each site was maintained at +4°C for up to one week for microbiological analyses and at −20°C for molecular analyses until further laboratory processing.

### Physical and chemical properties of soil samples

The following soil physical-chemical parameters were measured according to the Hungarian soil standard procedure: the texture of soils was loam according to the USA method. Humus content (g kg^−1^ soil) was calculated as soil organic C (g kg^−1^ soil)×1.725. Total salt content was measured from the electric conductivity (EC 2.5) of soil: water (1:2.5, [w/w]) suspensions. pH_(H2O)_ and pH_(CaCl2)_ (0.01 M), NH_4_^+^-N (mg kg^−1^), NO_3_^−^-N (mg kg^−1^), total N (g kg^−1^), and ammonium-lactate (AL)-soluble nutrient contents: Ca (g kg^−1^), K_2_O (mg kg^−1^), Na (mg kg^−1^), and P_2_O_5_ (mg kg^−1^) were assessed. Macro- and microelements were measured by an inductively coupled plasma atomic emission spectrometer (ULTIMA 2 ICP Optical Emission Spectrometer, Jobin Yvon Technology, HORIBA France SAS, Montpellier, France) and the soluble nutrient content with Lakanen-Erviö extractant was calculated (15, 16).

### Colony-forming units (CFU) of soil microorganisms

Ten-fold dilutions of soil suspensions were prepared in sterilized tap water. One hundred microliters of each dilution was used for the inoculation. Three plates were used for each dilution. To assess the CFU of spore-forming bacteria, the dilution series were heat treated at 90°C for 10 mins before spreading onto the medium. The number of heterotrophic bacteria and spore-forming bacteria (CFU) were measured on nutrient agar (Merck Millipore, Germany), and that of fungi on Rose Bengal Chloramphenicol agar (Merck Millipore) after a 5-d inoculation period at 25°C.

### Total enzyme activity of the soil biota

A fluorescein diacetate (FDA) hydrolysis assay was used to measure total soil microbial activity (74), modified by Adam and Duncan (1).

### Basal- and substrate-induced respiration

The activity of soil microorganisms was measured with basal (BRESP)- and substrate-induced respiration (SIR) techniques (6). The experiment was based on the CO_2_ evolution of 3–3 parallel measurements per soil sample using gas chromatography (GC 8000, Fisons, Rodano, Italy) with Clarity 4.0 software (DataApex, Prague, Czech Republic). Regarding BRESP, evolved CO_2_ was measured after 4 and 24 h and the difference between them gave the rate of CO_2_ production (μg CO_2_-C g soil^−1^ h^−1^). Regarding SIR, 200 μL D-glucose in 80 g L^−1^ solution was added to 2.0 g soil in a 27-cm^3^ vial. Evolved CO_2_ was measured after 3 h. The incubation for BRESP and SIR was performed in a water bath with shaking (25°C, 60 rev min^−1^). The initial concentration of CO_2_ in lab air was also recorded and considered as the starting point for measurements (11, 52). SIR values (μg CO_2_-C g soil^−1^ h^−1^) were then calculated as the difference between the end and initial concentrations, divided by the mass of the sample and time (80).

### Metabolic activity pattern of soil microbial communities

The MicroResp^™^ technique was used to evaluate the catabolic activity pattern of the microbial community of soils (17). Twenty-three different substrates and ultrapure distilled water (control) in four replications (subsamples per plate) were distributed to each plate. Four plates (*n*=4) were used for both OF and CF. The following substrates were used: D-galactose (Gal), trehalose (Tre), L-arabinose (Ara), D-glucose (Glc), and D-fructose (Fru) in 80 g L^−1^, citric acid (Cit), DL-malic acid (Mal), Na-succinate (Suc), L-alanine (Ala), and L-lysine (Lys) in 40 g L^−1^, L-glutamine (Gln) in 20 g L^−1^, L-arginine (Arg), 3.4-dihydroxybenzoic acid (Dhb), and L-glutamic acid (Glu) in 12 g L^−1^, myo-inositol (Ino), D-xylose (Xyl), D-mannitol (Mat), D-mannose (Man), D-sorbitol (Sor), and L-rhamnose (Rha) in 80 g L^−1^, L-asparagine-monohydrate (Asn) in 20 g L^−1^, and D-gluconic-acid-potassium (Gla), and L-ascorbic acid (Asa) in 40 g L^−1^. The pH of these substrate solutions were adjusted to 6.5 by 1 M NaOH or HCl solutions. In colorimetric detection, plates were read before and after 6 h of incubation at 25°C with a plate reader (Anthos 2010; Biochrom, Cambridge, UK) at 570 nm. Respiration rates were then calculated from normalized % CO_2_ data after 6 h, as described by the manufacturer (17).

### MPN pot experiments

Soil samples from OF and CF plots were tested on pea (*Pisum sativum* L.) and spelt wheat (*Triticum spelta* L.) host plants with different AMF dependence in an MPN experiment (62). The MPN bioassay enumerates all possible propagules (vesicular, viable soil-or root-born spores, hyphae infected roots), which have the ability to colonize the host plant.

The soil samples used for inoculation were diluted in pumice: 1× (100 g soil+200 g pumice per pot), 10×, 100×, 1,000×, and 10,000× (*n*=5). Non-inoculated plants were used as the control without soil. Germinated seeds (1 seed per pot) were planted into 350-cm^3^ pots containing pumice medium with a 0.6–1.0-mm particle size, pH of 6.5, 0.94 kg L^−1^ bulk density, and 0.26 cm^3^ per cm^3^ water content at field capacity. Pumice was AMF-free. In the estimation of the colonization potential of soils, pot cultures of infected peas or spelt wheats were grown for two months in a growth chamber under controlled climate conditions (26/18°C and 16/8 h for day/night and 600 μmol m^2^ s^−1^ photon flux density). Planting medium was irrigated daily with tap water to field capacity. The optimal plant nutritional status was maintained by weekly irrigations with modified Hoagland’s solution (50 mL pot^−1^ with only 0.1 M KH_2_PO_4_). The pot experiment was evaluated after an 8-week growing period by gently removing the roots from all pots.

### Estimation of root nodulation

An ordinal scale was introduced to visually estimate the number of nodules on the root systems: 0, no nodules at all, 1, only some nodules are visible (<10), 2, a few (>10) nodules on roots, 3, nodulation on a medium level (>40), 4, many nodules, the root system is fully nodulated (>100).

### Isolation of bacteria from nodules

In order to isolate rhizobia from the nodules of pea plants, pots containing the 10× dilution of original soils were selected (*n*=5). Three-five red root nodules per pot were examined, surface sterilized, and used to inoculate YMA agar (yeast-extract mannitol agar with Congo red) in order to isolate symbiotic nitrogen-fixing bacteria (84). After the inoculation, plates were incubated at 28°C for 5 d. Fifty-three randomly selected colonies were isolated from YMA plates. DNA was extracted from purified isolates (30, 84).

### Genetic diversity of isolated root nodule bacteria

The BOXA1R primer (5′-CTACGGCAAGGCGACGCTGACG-3′) (47) was used for BOX-PCR targeting the BOX-repetitive element (51, 82). The PCR amplification of DNA was performed in 50 μL containing 100 μM of the BOXA1 primer, 24 μL Dream Taq Green 2× Master Mix (Thermo Fisher), 24 μL nuclease-free water, and the DNA extract. PCR amplifications were performed in a thermal cycler (ICYCLER Thermal Cycler, Bio-Rad Laboratories, Hercules, CA, USA) with an initial denaturation (95°C, 2 min) followed by 35 cycles of denaturation (92°C, 30 s), annealing (50°C, 1 min), and extension (65°C, 8 min) with a final extension (65°C, 8 min). Each PCR product was analyzed by electrophoresis on 1.5% agarose gels. DNA extracted from *Rhizobium leguminosarum* strain RhPs 10 from the microbial strain collection of the Institute for Soil Sciences and Agricultural Chemistry (30) was used as a positive control.

### Estimation of AMF root colonization

Root samples from the pots were cleared and stained with acid glycerol aniline blue (61). Root segments were examined using an Olympus BX51 microscope (Tokyo, Japan) at a magnification from 40× to 200×. Frequency (F%), the intensity (M%) of mycorrhizal colonization, and absolute arbuscular richness (A%) as signs of AMF infectiveness were estimated by rating the density of colonization on 30-cm root segments using the five class system (81). The frequency of DSE-colonized roots was assessed with the same method. The MPN of AMF propagules was quantified using a bioassay that provides the number of infective propagules per unit soil weight (3).

### Molecular diversity of AMF

The AMF species colonizing plant roots in the MPN test (concentrated–100 g soil in pumice) were identified by sequencing the partial 18S-ITS1–5.8S-ITS2 rDNA region, as described by (65), with minor modifications. Randomly selected root pieces with a total length of 20 cm were crushed with pellet pestles in 1.5-mL microcentrifuge tubes. DNA was extracted and purified using the Qiagen DNeasy Plant Mini Kit (Qiagen, Hilden, Germany) according to the manufacturer’s instructions. Extracts were diluted 1:10 before PCR. The primers NS5/ITS4 were used in the first step of nested PCR (66) with an annealing temperature of 51°C. Before the second amplification, PCR products were diluted 1:100, and were then amplified with the primers GLOM1310/ITS4i as described by (65) and (66) with an annealing temperature of 61°C for 31 cycles. PCR products of the right size were cloned by the pGEM-T vector system and ligation mixtures were transformed into JM109 competent cells (Promega, Madison, Wisconsin, USA). To check for the presence of the inserts, successful clones were amplified, and 4 μL of the PCR products were then digested with the restriction enzymes MboI and HinfI (Fermentas, Vilnius, Lithuania) in 15 μL at 37°C overnight. Restriction fragment patterns were run on a 3% gel made from SeaKem LE Agarose (Lonza, Basel, Switzerland) and analyzed by Phoretix 1D software (Nonlinear Dynamics, Durham, NC, USA). A representative clone for each distinct restriction profile was amplified again by the nested PCR second step and further cleaned by the E.Z.N.A. Cycle Pure Kit (Omega Bio-Tek, Norcross, Georgia, USA). Purified products were sequenced using the BigDyeTM Terminator Cycle Sequencing Kit (Applied Biosystems, Foster City, CA, USA) and an automatic sequencer (ABI PRISM 3100 Genetic Analyzer; Applied Biosystems). AMF sequences were submitted to the BLAST query tool (5) for an initial similarity analysis. After removing chimeric results, 10 sequences were obtained and deposited in the EMBL and NCBI databases under the accession numbers KX463675 to KX463684.

### Statistical analysis

Significant differences in soil chemical, physical, and microbial properties (*n*=12), BRESP and SIR, and MicroResp^™^ (*n*=4) (every third sample was selected from a total of 12 samples along the diagonal line throughout the plot) measurements between the OF and CF types were tested by two sample *t*-tests. The SIMPER test was used to identify which physical-chemical parameters had the largest contribution to the average dissimilarity of MicroResp^™^ profiles between OF and CF. Since ascorbic acid showed a markedly high CO_2_ respiration value, it was excluded from further analyses. PCA was used to compare soil chemical parameters with OF and CF. RDA was performed to investigate the relationship between catabolic activity profiles (MicroResp^™^) and soil chemical, textural parameters. A regression using backward selection (function ordistep) was used to select the most important environmental variables for ordination (*P*-value<0.02). The Mantel test was utilized to compare the two land management sets of dissimilarities in the MicroResp^™^ profile based on Pearson’s product-moment correlation by a permutation test with the R i386 3.2.2. program (https://www.R-project.org/ [accessed 17.03.07]) using the vegan package 2.4-2 (https://cran.r-project.org/ [accessed 17.03.07]).

The BOX-PCR profiles of *Rhizobium* isolates assuming a 95% similarity threshold were analyzed by the TotalLab TL120. v 2006 software. Shannon, Margalef, and Pielou indices (45, 48) were calculated to characterize genetic diversities.

A phylogenetic analysis of AMF was performed by a distance analysis using the neighbor-joining method in PAUP 4b10 (79) with the Kimura two-parameter model, and a gamma shape parameter of 0.5. Bootstrap analyses were performed with 1,000 replications. Sequences selected from public databases belonging to known AMF species were included in the phylogenetic analysis. The effects of agricultural practices on root nodulation and the colonization of root-associated fungi in the MPN test were examined by ANOVA and LSD.

## Results

### Physical and chemical properties of soil samples

**Fig. 1:**
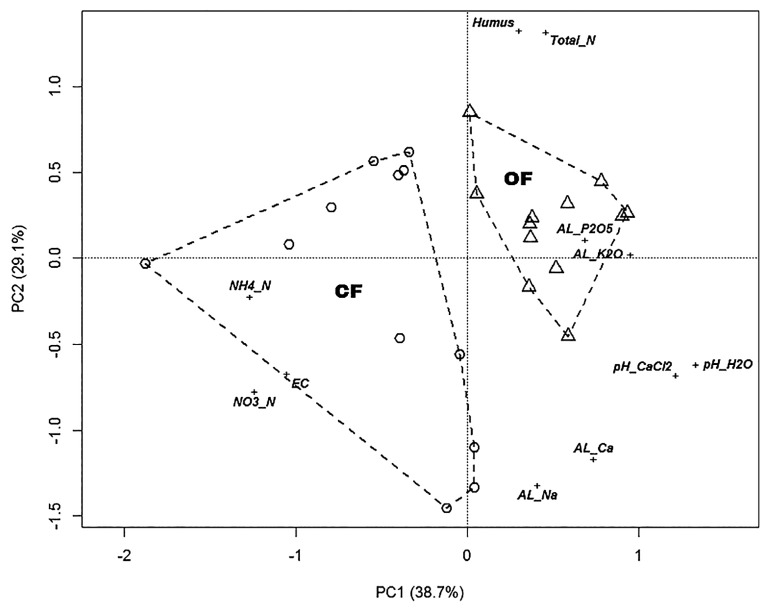
Ordination biplot of a principal component analysis (PCA) of soil chemical properties of soil samples from organic (OF) vs. conventional farming (CF) systems. Data are reported as avg, *n*=12. Legends: circle=soil from CF, triangle=soil from OF, EC=electric conductivity, total N=total nitrogen, AL=ammonium-lactate (AL)-soluble nutrient contents.

The pH_(H2O)_, pH_(CaCl2)_, total N values, AL-K_2_O, and humus content of soils were significantly higher in OF than in CF. However, EC, NH_4_^+^-N, and NO_3_^−^-N were significantly lower in CF than in OF. No significant differences were observed between the particle size distribution and AL-Ca, AL-P_2_O_5_, or AL-Na (Table 1). The results of the PCA analysis (Fig. 1) showed that OF and CF were completely separate from each other. The NO_3_^−^-N, EC, and NH_4_^+^-N parameters increased in the direction of CF, while pH(CaCl_2_), pH(H_2_O), AL-K_2_O, AL-P_2_O_5_, total N, and humus parameters increased in the direction of OF.

Soil management history primarily resulted in higher concentrations of the following nutritional elements: B, Ba, K_2_O, Mg, Zn, As, S, and Sr, in OF management than in CF, and soils under CF had significantly higher Al, Cr, Fe, and Ni contents. Differences in soil Ca, Cd, Co, Cu, Mn, Mo, P_2_O_5_, Pb, and Sn contents were not significant (Table S B1).

### Soil microbe counts and activities

Spore-forming, heterotrophic bacteria and filiform fungus CFU values as well as FDA, SIR, and BRESP data were significantly higher in OF than in CF (Table 2).

### Soil catabolic activity

**Fig. 2:**
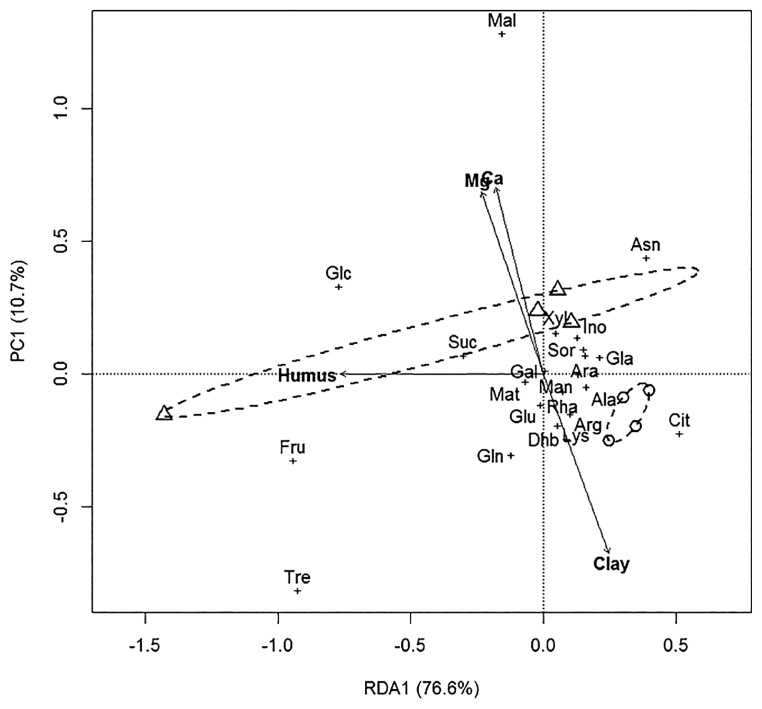
Relationships between catabolic activity profiles of organic (OF) vs. conventional (CF) management using RDA Circle=soil from CF; triangle=soil from OF; (Humus)=soil humus; (Ca)=calcium and (Mg)=magnesium contents of soil samples; (Clay)=clay percentage; Gal=D-galactose; Tre=trehalose; Ara=L-arabinose; Glc=D-glucose; Fru=D-fructose; Cit=citric acid; Mal=DL-malic acid; Suc=Na-succinate; Ala=L-alanine; Lys=L-lysine; Gln=L-glutamine; Arg=L-arginine; Dhb=3.4-dihydroxybenzoic acid; Glu=L-glutamic acid; Ino=Myo-inositol; Xyl=D-xylose; Mat=D-mannitol; Man=D-mannose; Sor=D-sorbitol; Rha=L-rhamnose; Asn=L-asparagine-monohydrate; Gla=D-gluconic-acid-potassium; Asa=L-ascorbic acid. The constraining factor was humus.

RDA separated samples to their own group based on different substrate utilization patterns, and the types of land use were also clearly separated from each other (Fig. 2). The microrespiration catabolic activity of OF was higher than that of CF (Fig. SA1). Significant differences in the microbial catabolic activity of OF and CF were detected. According to the SIMPER test, the most differentiating substrates between OF and CF were Glc, Fru, Mal, Gla, and Cit (*P*<0.05). Soil microbial activity to citrate was significantly higher (*P*<0.05) in CF than in OF, while that to malic acid was significantly higher in OF than in CF. The Mantel test showed a Pearson’s correlation coefficient of R=0.32 (*P*=0.002) by comparing OF and CF.

### Genetic diversity of nitrogen-fixing bacteria

Fifty-three pink nodules were collected from OF (17 nodules), CF (17 nodules), and control (19 nodules) treatments (in the MPN test). Control plants may have been nodulated due to the microbes inhabiting the seed surface. Differences in BOX-PCR patterns were indicated among OF, CF, and control plots. Shannon and Margalef diversity indices of *Rhizobium* sp. isolates from OF samples were higher (2.15; 3.18) than those from CF (2.02; 2.95) and control (1.98; 2.95) samples (Fig. SB), while the Pielou index was markedly higher for strains isolated from CF pots (0.92) than those from OF (0.76) and control pots (0.73).

### Symbiotic efficiency of nitrogen-fixing bacteria

**Fig. 3:**
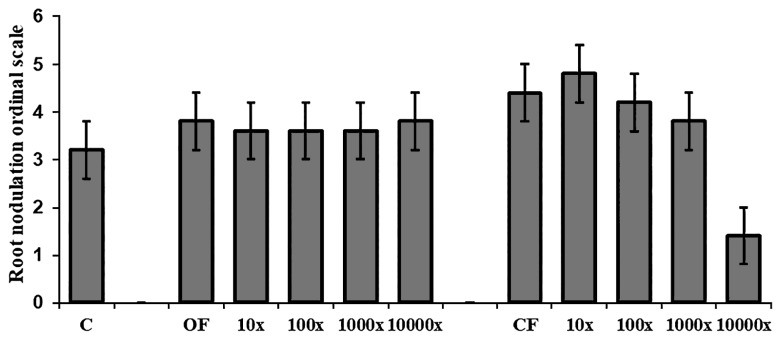
Root nodulation ordinal scale of pea plants from pot experiments with soils from organic and conventional fields of Martonvásár diluted in pumice. Data represent the sum of ordinal values estimated for central and lateral roots. (Legends: avg±LSD; (*n*=5); C=control (pumice), 1×–10,000×–dilution series).

Pea plants were infected by *Rhizobium* bacteria in OF and CF samples (Fig. 3). Control plants were infected by microsymbionts originating from the seed surface, and, similar to pumice, no indigenous *Rhizobia* were detected. No significant differences were observed between the nodule number of pots with OF and CF samples and the control. Regarding pea plants from OF, soil without dilution (1×) showed a markedly higher number of nodules (3.80±0.44) than the members of the dilution series.

The 10× soil dilution had the highest number of nodules (4.80±0.44) at conventional land management. The number of nodules decreased with increasing soil dilutions. Nodule number reductions with soil dilution were only observed in pots with soil from CF.

### Number of AMF infective propagules (IP) and AMF diversity; occurrence of DSE

Microscopic observations of stained roots from control pots showed the absence of fungi. The number of AMF IP on pea roots from OF (peaMPN_OF_=70 propagules g^−1^ dry soil) was one order of magnitude higher than those from CF (peaMPN_CF_=3.3 propagules g^−1^ dry soil). The highest colonization rate (meanM_pea_%) in pots inoculated with soil from OF was 60%, while that in pots inoculated with soil from CF was only approximately 30% in the case of the 10× dilution. The M% of pea roots from both cultivation methods showed slight decreases along the dilution series. The extent of M_pea_% in roots ranged between 0.07 and 88.17%. In 1×-100× dilutions of OF soil, the intensity of pea mycorrhizal colonization was significantly higher than in the same dilutions of CF soil.

The opposite results, apart from lower colonization rates, were observed in spelt wheat for the number of AMF IP. The MPN of CF soil using the spelt wheat host (speltMPN_CF_=4.9 propagules g^−1^ dry soil) was slightly higher than in OF soil (speltMPN_OF_=3.3 propagules g^−1^ dry soil). The highest colonization rate (meanM_spelt_%) in pots inoculated with soil from CF was 25%, while that in pots inoculated with soil from OF was approximately 10%. AMF root colonization indicated a higher susceptibility of pea than spelt wheat for indigenous AM fungi.

Fungi with the morphology of DSE were detected in soils of the CF system only ([F%] of colonized roots=20%). Root length colonization by DSE fungi negatively correlated with AM fungal colonization. Two groups of AMF sequences with a similarity to *Rhizophagus intraradices* or *Funneliformis mosseae* were identified from OF pea and wheat and CF pea roots (Fig. SC1). Organic peas had sequences from both groups (KRSZ 25, 26, 27), while conventional peas only had those from the *Rhizophagus* clade (KRSZ 20, 22, 23). Organic wheat only had *Funneliformis* sequences (KRSZ 13, 14, 16, 19).

## Discussion

Soil microbial communities are key components of soil services in agroecosystems that impact on the quality and quantity of food production, the efficiency of cropping systems (86), and the emission of greenhouse gases (7). Therefore, it is important to select a site-specific soil management practice that improves soil health (60). We investigated the influence of organic and conventional agronomic practices on the physical-chemical and microbiological properties of soils, including the most important functional groups of the soil biota in agricultural ecosystems. Indicators were selected in a system-oriented approach and their relationships were analyzed to characterize soil quality.

AL-K_2_O, pH_(H2O)_, pH_(CaCl2)_, total N, and humus contents were significantly higher under organic farming conditions (Table 1). Clark *et al.* (19) reported similar findings in organic fields after four years from a transition from CF. Clark *et al.* (19) found that inputs of 8,030 kg C ha^−1^ year^−1^, 142 kg P ha^−1^ year^−1^, and 166 kg K ha^−1^ year^−1^ were higher in the case of OF due to animal manure application and cover crop incorporation. Gosling *et al.* (34) showed a similar outcome for total N. Higher total N values may have been connected to high humus contents. In addition, OF increased the number of infective AM propagules in the soil. The lower humus values of CF plots may be related to the limited input of organic matter and the increased decomposition rate of organic residues due to mineral N addition satisfying the N requirements of microorganisms (12). The significantly higher NH_4_^+^-N and NO_3_^−^-N contents of CF soils may have been due to the improved control of soil N levels in OF soils by soil microbes and a higher chance of N loss through leaching or denitrification in CF soils.

In the present study, pH values were slightly alkaline (Table 1), which is consistent with previous findings, because increasing soil pH was demonstrated after 5–10 years of OF (22, 25, 49). Chemical fertilizers provoke soil acidification, which may have contributed to the significantly lower pH of the CF field. The level of biological activity changed very slowly in response to fertilization and cultivation techniques; therefore, differences in microbial activity and enzyme activity between OF and CF plots have rarely been observed in short-term on-farm investigations (32, 50). In our long-term study, the activity of soil microbes was already significantly higher in organically managed plots (Table 2). Activated soil microorganisms play a crucial role in the decomposition of soil organic matter and the release of stored nutrients, *e.g*. N or P, improving the supply of plants (35).

According to the SIMPER test, the largest contribution to the dissimilarities of catabolic profiles was attributed to glucose (Fig. SA1) (also indicated by [69]), fructose, malic acid, and citric acid (*P*<0.05) in decreasing order of importance.

Campbell *et al.* (17) indicated that the above carbon sources (as root exudates) have a large impact on soil ecological processes. In our experiments, the catabolic activity of OF soil was significantly higher than that of CF soil. PCA analyses of soil chemical properties and an RDA analysis of soil metabolic activity patterns showed two significantly separated functional groups from the different land management systems.

A dendrogram of the BOX-PCR profiles (Fig. SB) of nodule-inhabiting bacterial strains did not show a clear separation of the two land managements; however, Shannon and Margalef indices indicated a diversity benefit for OF soils, similar to previous findings (43, 45).

Our results on indigenous AMF root colonization are consistent with previous findings on the AM status of hosts in low-input soils: numerous laboratory and field studies demonstrated the negative effects of intensive soil management, such as chemicals, physical disturbances, and biotic variables on AM formation (34). Significant differences were observed between the richness of native AMF IP in soils collected from OF and CF systems (34). The intensity of AMF root colonization in pots inoculated with soil from OF was also high, suggesting that the mycorrhizal status of plants growing in these soils correlated with the level of IP. Mäder *et al.* (48) found that the percentages of AMF colonized root length were 30–60% higher in the roots of winter-wheat (*Triticum aestivum* L. cv. Sardona), vetch-rye, and grass-clover grown in soils from organic or bio-dynamic low-input farming systems than in plants grown in high-input conventionally farmed soils.

In both plants, the highest level of AM colonization (M%) was noted in the 10× dilution of soil, presumably due to weaker phosphorus nutrition promoting colonization, which was compensated by the addition of larger quantities of high-nutrient soil supply in the case of the concentrated pots. The MPN of AMF in spelt wheat was not significantly different between OF and CF. The pea crop was more suitable for examining the maximum number of propagules due to higher AMF colonization.

A phylogenetic analysis (Fig. SC1) revealed only the dominant clades of AM fungi of the *Glomeraceae* family (46), which is the most highly represented group in similar studies of agricultural fields (27). It is intriguing and also in parallel with the results of this study that both *Rhizophagus* and *Funneliformis* sequences were only found in the organic pea samples, while conventional wheat samples yielded no AMF sequences.

Previous studies (57, 58) showed that AMF biodiversity was higher in low-input systems than in high-input systems, although this finding has not always been unambiguous (27, 37, 83). Another study (26) found only a negligible effect on fungal communities after 15 years of low input agricultural practice. Sasvári *et al.* (72) assessed the diversity of AM fungi colonizing maize roots grown in a long-term monoculture experiment established in Martonvásár, Hungary. A phylogenetic analysis assigned the *Glomeromycota* sequences into 22 operational taxonomic units belonging to the *Archaeosporaceae*, *Glomeraceae*, and *Paraglomeraceae* families. Similar to the present results, *Glomus* group A fungi dominated in the AMF community of maize. They highlighted the importance of efficacy differences among similar communities in terms of improvements in plant nutrition and growth. These findings show that the biodiversity of AMF communities is not always low in intensively managed systems. The colonization and effects of AMF or DSE root-associated fungi on plants vary and may depend on the host species, fungal taxa or strains, and environmental conditions. It has been hypothesized that endophytic fungi are latent pathogens, while others concluded that symbiosis with DSE changes between mutualism and parasitism in fine degrees, similar to mycorrhizas (39). The symbiotic role of DSE in host-fungal associations is supported by studies on host responses to a DSE inoculation and its frequent occurrence in abiotic-stressed habitats (64, 87). Ours is the first detailed study on the DSE status comparing the effects of OF and CF on DSE occurrence. DSE occurrence in intensively managed fields indicated that the habitat of the microbial community lost its balance due to the presence of stress conditions.

Biomass, genetic diversity, and the activity of soil biota, as well as its effects on the productivity of soils and their ability to alleviate environmental constraints and stress factors are all important properties to consider when evaluating soil quality. Therefore, instead of a pre-selection of parameters, the present study aimed to apply an extended minimum data set of classical status and functional tests to acquire a complex view of soil microbes. Our results corroborated previous findings showing that organic management improved the different parameters of soil quality, *e.g*. nutrient status, biomass, and the diversity of indigenous symbiotic soil microbial populations or enzyme activities. When comparing organic versus conventional managements effects on the activity and composition of soil microbial communities, soil type and vegetation must be also considered as they have significant influence on them. In many cases, organically managed plots are set up on soils already in a better condition, which precludes a robust change afterwards.

Some of the biological system-oriented indicators, *e.g*. rhizobia, may react markedly faster than AM fungi, which showed less pronounced differences even after several years of different management. During complex investigations, it is important to weigh the measured parameters appropriately for an adequate evaluation and identify those that may really indicate a significant shift from the quality status of conventionally managed soil. Based on this information, habitat- and soil-specific protocols need to be implemented for practical interventions to sustainably and efficiently improve soil conditions.

## Supplemental material



## Figures and Tables

**Table 1:** Main soil physical and chemical properties of organic (OF) and conventional fields (CF) of Martonvásár

Soil physical-chemical properties^a^	Martonvásár farming system		

	OF	CF	LS^b^
Sand (%) (0.05–2 mm)	32.7±3.2	30.1±4.5	n.s.
Silt (%) (0.002–0.05 mm)	42.4±2.6	43.0±2.7	n.s.
Clay (%) (<0.002 mm)	24.9±1.3	27.0±2.8	*
AL-Ca (g kg^−1^)	1.6±0.5	1.2±1.0	n.s.
AL-P_2_O_5_ (mg kg^−1^)	737±261	654±46.1	n.s.
AL-K_2_O (mg kg^−1^)	605±89.3	526±58.2	**
AL-Na (mg kg^−1^)	11.2±1.0	11.1±3.2	n.s.
EC (dS m^−1^)	0.2±0.0	0.3±0.1	**
pH_(H2O)_	8.0±0.1	7.8±0.2	**
pH_(CaCl2)_	7.9±0.1	7.7±0.1	**
NH_4_^+^-N (mg kg^−1^)	4.9±0.7	8.8±5.0	**
NO_3_^−^-N (mg kg^−1^)	5.2±1.0	40.2±17.9	***
total N (g kg^−1^)	1.9±0.0	1.7±0.0	***
humus (g kg^−1^)	3.0±0.3	2.7±0.2	**

Data are reported as avg±SD of the means, *n*=12. Legend:

a EC=electric conductivity; total N=total nitrogen; AL=ammonium-lactate (AL)-soluble nutrient content.

b Significant differences among different land managements, two sample *t*-tests, P*=<0.05; P**=<0.01; P***=<0.001; n.s.=not significant between OF and CF; level of significance=LS).

**Table 2:** Soil microbe counts and activities of organic (OF) and conventional fields (CF) of Martonvásár

Soil properties	Unit	Martonvásár farming system		

		OF	CF	LS^b^
CFU_s_	Log CFU g^−1^ dry weight soil	5.5±0.1	5.0±0.2	***
CFU_h_		8.3±0.1	7.8±0.5	***
CFU_f_		5.7±0.1	5.2±0.2	***
FDA	μg Fl g^−1^ soil h^−1^	47.8±6.4	24.0±0.1	***
BRESP	μg CO_2_-C g^−1^ soil h^−1^	0.8±0.2	0.4±0.1	*
SIR		7.7±1.0	3.9±0.5	***

Data are reported for CFU and FDA as avg±SD of the means, *n*=12; for BRESP, SIR *n*=4. s=spore-forming bacteria; h=heterotrophic bacteria; f=fungus; FDA=fluorescein diacetate hydrolysis; Fl=fluorescein diacetate; BRESP=basal respiration; SIR=substrate-induced respiration.

b Significant differences among OF and CF, two sample *t*-tests, P*=<0.05; P**=<0.01; P***=<0.001; n.s.=not significant between OF and CF; level of significance=LS.
