# Supplementary material for: Density and Diversity of Microbial Symbionts under Organic and Conventional Agricultural Management
Source: Microbes Environ. 2019 Jun 13;34(3):234–43. doi: 10.1264/jsme2.ME18138 (PMC6759338; doi:10.1264/jsme2.ME18138)

Supplemental material

Table S A1
Crop rotation management and fertilization of arable farming types in organic and conventional systems in the 5 years before sampling.

Years	OF[a]		CF[a]	
	Crop	Fertilization	Crop	Fertilization[b]
2008	cereal	no	corn	300 kg ha^{-1} NPK (15:15:15%)
				270 kg ha^{-1} N (39%)
2009	corn	no	spring wheat	300 kg ha^{-1} NPK (15:15:15%)
				400 kg ha^{-1} N (39%)
2010	oil radish	green manure (oil radish)	corn	300 kg ha^{-1} NPK (15:15:15%)
				300 kg ha^{-1} N (39%)
2011	peas	green manure (peas)	spring wheat	250 kg ha^{-1} NPK (0:10:24%)
2012	cereal	no	corn	300 kg ha^{-1} NPK (15:15:15%)
				270 kg ha^{-1} N (39%)

[a]OF=organic farming; CF=conventional farming system. [b]Complex NPK fertilizer was used with 15-15-15% active agent. 15% N:10% ammonium nitrogen +5% urea N; 15% P2O5:P content 6.2%; 15% K2O:K content 12.5%), N fertilizer (calcium ammonium nitrate fertilizer (CAN)) was used which contain 27% N, 5% Ca (7% calcium oxide) and 3% Mg (5% magnesium oxide)).

Table S B1
Nutrient and macro- and micro elements content of organic (OF) and conventional fields (CF) of Martonvásár.

[a]	Martonvásár farming system		
(mg kg^{-1})	OF[b]	CF[b]	LS[c]
Al	57.7±21.1	86.7±26.0	**
As	0.3±0.1	0.2±0.1	*
B	2.2±0.2	1.7±0.1	***
Ba	27.4±1.4	21.4±2.7	***
Ca	15420±4940	11440±8630	n.s.
Cd	0.1±0.0	0.1±0.0	n.s.
Co	2.8±0.5	3.3±1.0	n.s.
Cr	0.06±0.0	0.08±0.0	***
Cu	5.9±1.1	5.6±1.5	n.s.
Fe	74.2±7.0	107±23.1	***
K_2O	594±90	531±57	*
Mg	587±79	426±49	***
Mn	389±59	426±100	n.s.
Mo	0.02±0.02	0.01±0.01	n.s.
Ni	3.7±0.9	4.9±1.6	*

P_2O_5	451±200	426±67	n.s.
Pb	7.7±1.1	7.4±1.6	n.s.
Sn	0.03±0.06	0.02±0.05	n.s.
Sr	26.9±5.6	20.4±9.0	*
Zn	5.7±1.3	3.5±1.0	***
S	18.4±1.9	15.6±3.8	*

Data are reported as (avg±SD of the means, n=12). [a]Nutrient and macro-micro elements content of soils. [b]OF=organic farming; CF=conventional farming system. [c]Significant differences among different land managements, two sample paired tests, P*=<0.05; P**=<0.01; P***=<0.001; n.s.=not significant between OF and CF; LS=level of significance.

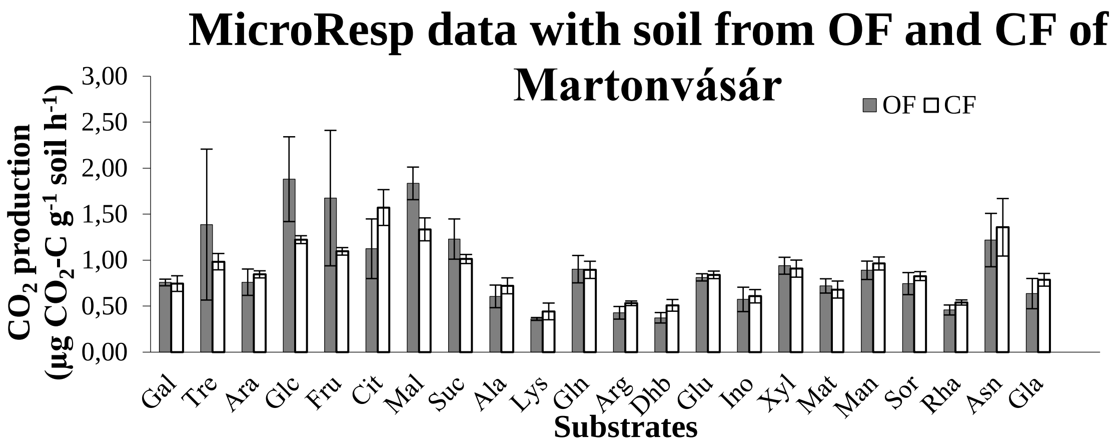

Fig. S A1. CO_2 production of the soil samples from the organic (OF) and conventional farming (CF) of Martonv§s§r. (Legend: avg±SD of the means; n=4; Gal=D-galactose; Tre=trehalose; Ara=L-arabinose; Glc=D-glucose; Fru=D-fructose; Cit=citric acid; Mal=DL-malic acid; Suc=Na-succinate; Ala=L-alanine; Lys=L-lysine; Gln=L-glutamin; Arg=L-arginine; Dhb=3.4-dihydroxybenzoic acid; Glu=L-glutamic acid; Ino=Myo-inositol; Xyl=D-xylose; Mat=D-mannitol; Man=D-mannose; Sor=D-sorbitol; Rha=L-rhamnoze; Asn=L-asparagin-monohydrate; Gla=D-gluconic-acid-potassium; Asa=L-ascorbic acid.

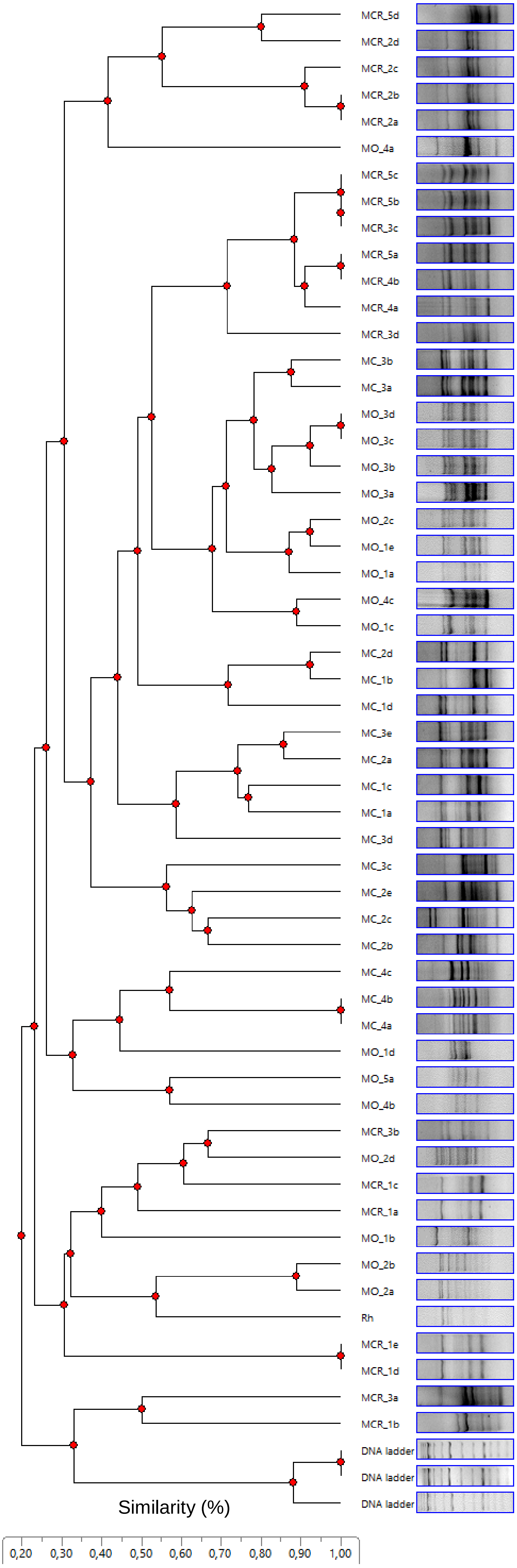

Fig. S B. Dendrogram of *Rhizobium* sp. isolates from organic, conventional and control plot of Martonv§s§r.
The homology dendrograms and diversity indices of *Rhizobium* strains isolated from the root nodules of pea plants from pot experiment from organic, conventional and control plot. Dendrogram with homology coefficient %: 1.0.
Legend: MO=organic; MC=conventional; MRC=control pots from Martonv§s§r; Rh=*Rhizobium leguminosarum* strain; DNA ladder=Gene Ruler 1 kb plus; lanes represent the BOX-PCR products with BOXA1 primer; numbers 1-5 indicates the technical replicate; the letters from a, b, c, d, e are show the number of nodules per pot; %=percentage of similarity.

Fig. S C1.

Phylogenetic analysis of arbuscular mycorrhizal fungi of roots from the MPN test.

Legend: Neighbour-joining phylogenetic analysis of arbuscular mycorrhizal fungal 18S rDNA sequences obtained from MPN root samples for *Glomeraceae*, rooted to *Paraglomus occultum* sequences. The scale represents substitutions per site. Sequences obtained in the present study are shown in boldface, including internal identification numbers (KRSZ). Sequences from databases isolated from spores were labelled with fungal species and accession number. Bootstrap values from 1.000 replications larger than 50% are indicated above branches.

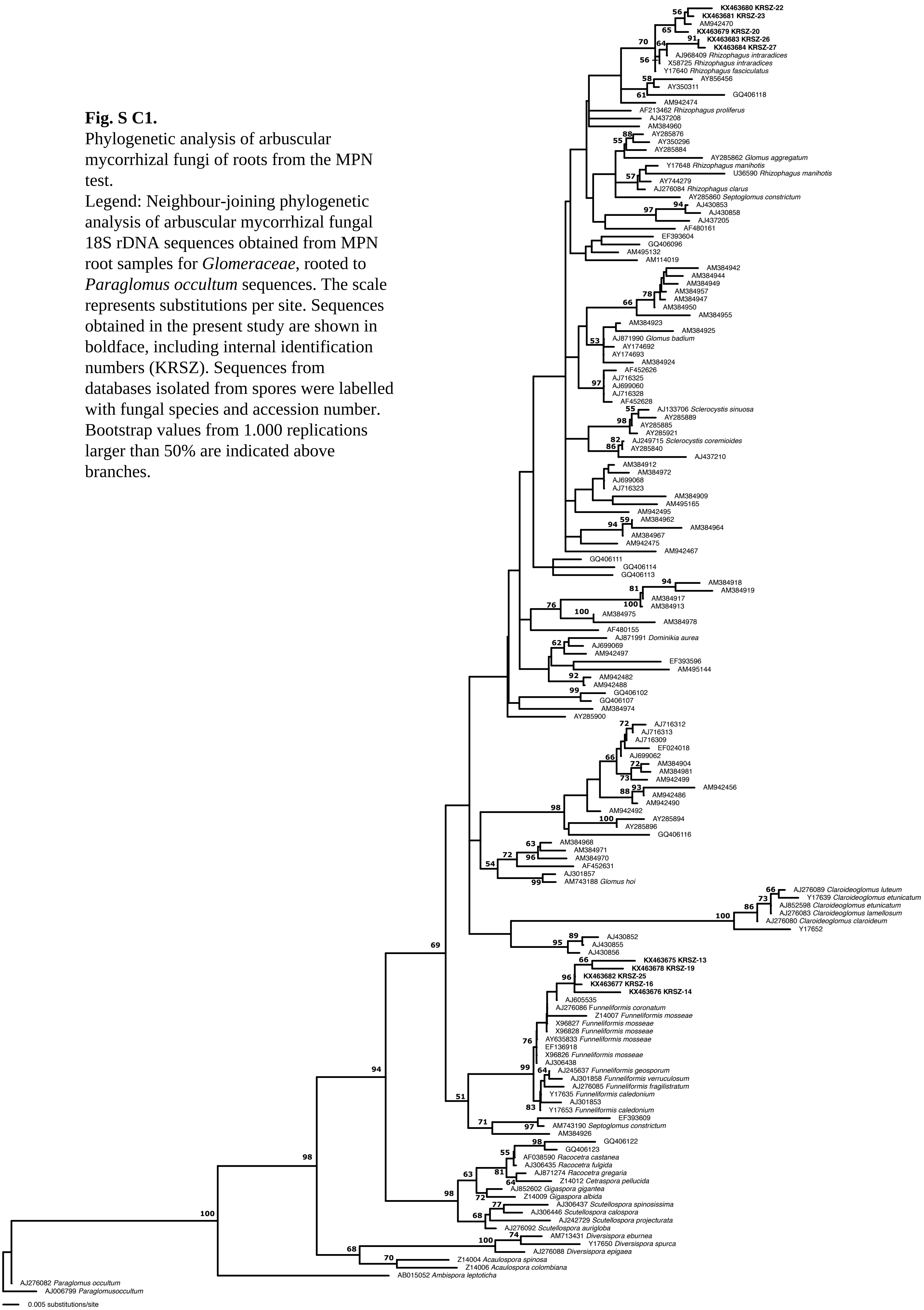

Supplement: Supplementary file 1 [file 34_234_s1.pdf]
